# Overcoming Distressing Voices: A Self-Help Guide Using Cognitive Behavioral Techniques

**DOI:** 10.1192/pb.bp.114.049502

**Published:** 2016-02

**Authors:** Deborah Cooper

This is a self-help book directed at those who experience voices. It is written in a format seen in a number of other previous self-help books. Its aims are simple – to increase understanding of the experience of hearing voices and reduce distress this causes.

The book is structured in five parts. The first part provides background knowledge about distressing voices by exploring topics such as the relationship between hearing voices and mental disorder, the common types of voices experienced by people and the relationship between voices and low self-esteem. The second and third parts provide strategies to reduce distress occurring as a consequence of voices. Examples include helping the reader to develop relaxation techniques and encouraging socialisation. This section also covers core aspects of cognitive-behavioural therapy such as identifying core beliefs and helping the reader to start to challenge these. The penultimate part encourages the reader to make plans for the future, and the last part explores the impact of voices on those close to the reader, such as friends and family.

The major strength of this book is its readable style. The introduction highlights that a number of individuals are reluctant to seek help regarding distressing voices. The narrative style and the effective use of case histories throughout add a personal feel to the book and perhaps help to reduce the sense of isolation that can occur as a consequence of hearing voices. A number of practical homework tasks are set. These help to support the reader to develop greater knowledge and awareness of their voice-hearing and develop the ability to manage distress.

A minor criticism of this book might be its relatively small dimensions. Although this makes it portable and approachable, the practical exercises contained within are on the small side. As a consequence, one would suspect that someone working through the book would need to keep some of the exercises separate from the book. This might make review of the knowledge and skills gained a little disjointed. The book is laid out clearly and logically, with short paragraphs that encourage the reader to work through short sections, as and when the opportunity arises, rather than reading the book in one go. However, I wonder if some readers might benefit from guidance in approaching the book, for example working alongside a carer or mental health professional.

On the whole, this book achieves its objectives. It would be of value to those suffering from distressing voices, as well as friends and family members who may seek greater understanding of these experiences.

